# Cannabis, cannabinoids and health: a review of evidence on risks and medical benefits

**DOI:** 10.1007/s00406-024-01880-2

**Published:** 2024-09-19

**Authors:** E. Hoch, N. D. Volkow, C. M. Friemel, V. Lorenzetti, T. P. Freeman, W. Hall

**Affiliations:** 1 Department of Psychiatry and Psychotherapy, LMU University Hospital, LMU Munich, Munich, Germany; 2 IFT Institut für Therapieforschung, Centre for Mental Health and Addiction Research, Munich, Germany; 3 Department Clinical Psychology and Psychotherapy, Charlotte Fresenius University, Munich, Germany; 4 National Institute on Alcohol Abuse and Alcoholism, Bethesda, Maryland USA; 5 Neuroscience of Addiction and Mental Health Program, School of Behavioural and Health Sciences, Faculty of Health Science, Australian Catholic University, Melbourne, Victoria 3065 Australia; 6 Addiction and Mental Health Group, Department of Psychology, University of Bath, Bath, UK; 7 National Centre for Youth Substance Use Research, Faculty of Health and Behavioural Sciences, The University of Queensland, Brisbane, Queensland Australia; 8 Queensland Alliance for Environmental Health Sciences, Faculty of Health and Behavioural Sciences, The University of Queensland, Brisbane, Queensland Australia

**Keywords:** Cannabis*/adverse effects, Cannabinoids*/therapeutic use, Humans, Medical Marijuana*, Marijuana abuse, Public health

## Abstract

The legalization of cannabis for medical and recreational purposes has progressed internationally. Cannabis and cannabinoids are advocated for a plethora of medical indications. An increasing number of medical and nonmedical users regularly consume large doses of delta-9-Tetrahydrocannabinol (THC), the main active component of cannabis. Aim: to summarize the evidence on (1) risks of recreational cannabis use and (2) effectiveness and safety of medicinal cannabis. Findings on recreational use: Cannabis is mostly used to experience its acute rewarding effects. Regular use of high THC products can produce addiction (cannabis use disorder or CUD). Acute consumption of high THC doses (including unintentionally) can cause time-limited mental, gastrointestinal, and cardiovascular problems and motor vehicle accidents. Chronic patterns of cannabis use have been associated with multiple adverse outcomes that are of particular concern among adolescents and young adults, such as, disrupted learning, impaired cognitive performance, reduced educational attainment and an increased risk of CUD, psychosis/schizophrenia, mood and anxiety disorders and suicidal behaviors. There is debate about the extent to which cannabis use is a cause of these adverse outcomes. Physical health risks (e.g., respiratory and cardiovascular, prematurity and restricted fetal growth, hyperemesis syndrome among others) have also been linked with repeated consumption of cannabis with a high THC content. Findings on medical cannabis use: Herbal cannabis, medicines from extracted or synthetized cannabinoids—often used as adjuvants to standard medicines—may produce small to modest benefits. This is primarily the case in treating chronic pain, muscle spasticity, chemotherapy-induced nausea and vomiting, and refractory epilepsy (in the case of cannabidiol, CBD). The evidence is inconclusive on their value in treating mental disorders and other medical conditions. Safety: Cannabis-based medicine is generally well tolerated. There is a risk of mild to moderate adverse effects and CUD.

**Supplementary Information:**

The online version contains supplementary material available at 10.1007/s00406-024-01880-2.

## Introduction

In 2021, the UNODC estimated that 219 million people, 4.3% of the global adult population, had used cannabis [1]. Its nonmedical use has been prohibited under international drug control treaties since 1961. Cannabis consumption remains the highest in North America, where 17.4% of the population aged between 15 and 64 used it in the past year [2]. Prevalence rates are lower in Oceania (12.2%), West and Central Africa (9.7%) and Europe (7.8%) [2] with considerable variations between countries within these regions. The past 25 years have seen different policies towards cannabis use for medical or recreational purposes [3]. In the United States and Canada cannabis legalization has been accompanied by increases in the number of regular users who consume increasingly higher doses of tetrahydrocannabinol (the main psychoactive ingredient in cannabis) [4, 5]. The higher potency of cannabis products [6], diverse cannabis formulations and modes of administration (e.g., edibles, beverages, vaping, and dabbing) have raised concerns about the adverse public health impacts of cannabis legalization particularly when there is little or no regulatory oversight [7].

We have also seen a rapid development in the basic science of cannabinoids, including characterization and function of the endocannabinoid system (ECS) in the human brain and other organs, the pharmacology of cannabinoids, and therapeutic cannabinoid development [8]. Growing evidence indicates that dysregulation of ECS may contribute to brain dysfunction, psychopathology, neurodevelopmental problems, neurodegenerative diseases, neuroinflammation, among other physiological functions [9, 10]. The last decade has also seen a significant increase in scientific literature addressing the effects of cannabis and cannabinoids for recreational and medicinal purposes [11–13].

To guide policies regarding access and oversight for cannabis for recreational of medicinal purposes it is crucial to summarize the literature on the risks for recreational use and its therapeutic benefits. In this review we summarize (1) mental, physical, and social risks of cannabis when used as recreational drug and (2) the efficacy and safety of cannabis-based medicines.

## Methods

This work aims to update a large systematic literature research on “benefits and risks of cannabinoids” commissioned by the German Ministry of Health [14–18]. For this reason, we conducted a selective literature research in PubMed and the Cochrane Central Register of Clinical Trials. The search period was from 10.5.2016 to 10.5.2023. PubMed was searched with combinations of the mesh terms “Cannabis” or “Cannabinoid” or “Marijuana” or “Medical Marihuana” or “Medical cannabis” or “Pharmaceutical cannabis”. Our search procedure gave priority to systematic reviews and meta-analyses of research on cannabis and cannabinoids, published in the past decade. In their absence, we included studies with the strongest research designs that had adequate statistical power and have been published since the most recent review. The references of the studies found were also screened to identify further relevant studies. We used the approach to causal inference outlined in Text Box 1.

**Text Box 1:** Approach to causal inferences [19]

In assessing whether cannabis was a cause of an adverse health effect we assessed
1. Evidence of an association between cannabis use and the health outcome
2. Evidence that reverse causation was an implausible explanation of the association from experiments (when ethically acceptable) and otherwise from prospective studies
3. Evidence that the association was not explained by uncontrolled and unmeasured factors, e.g., by statistical analyses controlling for confounding variables
4. Evidence that a causal relationship between cannabis use and the health outcome was biologically plausible from animal or human experiments, the neurobiology of the cannabinoid system and the pathophysiology of the health outcome

## Results

### Risks of recreational cannabis use

Acute effects may occur shortly after a single occasion or infrequent cannabis use. Chronic use is defined as daily or near daily use over months or longer.

#### Reinforcing effects

Cannabis is used recreationally because of its rewarding effects that are associated with decreased stress reactivity and an enhanced sense of well-being [11]. Rewarding affects can include positive effects e.g., euphoria and the relief of adverse experiences such as anxiety. These rewarding effects are linked with the agonist effects of THC at the cannabinoid CB1 receptor that drive THC’s modulation of dopaminergic signaling in the nucleus accumbens, the main reward center in brain. The anxiolytic effects of cannabis are mediated both by THC and cannabidiol (CBD) in part through their effects in the amygdala. The doses of THC and routes of administration determine the magnitude of its rewarding and anxiolytic effects. Other neurotransmitter systems, neuromodulators and individual metabolism differences also account for large differences in the effects of cannabis. At high THC doses, acute cannabis intoxication can produce unpleasant feelings (e.g. irritability, anxiety), distorted perceptions (e.g. paranoid thoughts, short-lived hallucinations, delusions or depersonalization) and dysfunctional behavior [20, 21]. Overdosing and unintentional cannabis use may cause short term psychiatric, gastrointestinal, and cardiovascular problems in children, teens, and adults [22, 23]. These unpleasant symptoms generally subside without the need for medical assistance but can require medical assistance in more severe cases.

#### Motoric incoordination

Acute cannabis consumption has consistently been associated with a modest increase in the risk of traffic accidents, with odd ratios ranging from 1.25 to 1.97 [17]. Measuring THC-related impairment and fitness to drive is more complicated than for alcohol because the absorption and bioavailability of THC does not follow a uniform time- and dose-dependent course and cannabis-related behavioral impairments differ from those of alcohol [24]. Thus, there are no reliable cut-off values for blood THC levels that indicate when an individual is impaired. Orally administered cannabis produces a protracted increase in blood THC concentration and prolongs impaired driving performance compared to inhaled cannabis. Frequent cannabis users may be less impaired because they may show tolerance to the motor incoordination and cognitive disruptive effects of cannabis. Recovery of driving-related skills appears to occur 5–6 h after cannabis intoxication but individuals may vary in the amount and duration of impairment [24]. The concurrent use of cannabis and alcohol produces greater driving impairment than cannabis alone [25].

#### Cognition

Acute intoxication with cannabis and cannabinoids (THC, THC/CBD extracts) increases disinhibition, and impairs verbal memory, other memory components, learning, attention, attentional bias, and psychomotor function [26, 27]. Acute intoxication has inconsistent effects on working memory, verbal fluency, executive function and time estimation.

Most of these effects recover within hours or a couple of days but they can persist for longer time periods in chronic cannabis users. In non-intoxicated, current cannabis users, impaired cognitive performance shows small-to-medium effect sizes [26, 27]. The domains most consistently adversely affected are forgetting (retrieval/recall) and learning, global cognition, memory and cognitive impulsivity [26, 27]. There is emerging evidence for decrements in specific memory components (i.e., verbal, encoding, episodic and prospective), visual recognition and attentional bias [26, 27]. Other cognitive domains that were inconsistently impaired include attention, executive function/abstraction, working memory/updating, psychomotor function, decision making, verbal/language, processing speed, verbal fluency, and other memory components [26, 27]. There were no differences in simple reaction time, visuospatial learning, visual immediate memory, recognition, attention (sustained, divided) and time estimation [26, 27]. There is preliminary evidence of cognitive impulsivity and poorer verbal memory and visual recognition in older studies of recreational users [28].

Cognitive alterations in cannabis users appear to improve with abstinence. For example, when abstinence lasted longer than 42 h, 72 h and 25 days, cognitive function improved in most domains [26, 29]. Impairments that were more persistent after abstinence in some studies included impulsivity, verbal memory and fluency, working memory, performance in other memory domains; learning; attention, executive function and psychomotor function.

Cognitive performance was generally not affected by age, although cognitive impairments were worse in older people with psychosis (e.g., cognitive impulsivity, verbal memory, visual recognition) [28]. There is mixed evidence on the effects of the age of first cannabis use and duration of use on cognitive performance, except for IQ, which decreased by 2 points each year after the start of frequent/dependent use [30]. Emerging evidence indicates that heavier use, higher doses and higher frequency of cannabis use adversely affect cognition [26, 29].

#### Motivation

There is partial support from longitudinal studies for a causal link between cannabis use and a reduction in motivation, that has been referred to as an “a motivation syndrome” [31]. It manifests as a reduce motivation to engage in everyday activities such as a diminished desire to study or work, engage in social or recreational activities people who use cannabis frequently [32]. A lack in motivation may also be related to decreased reward sensitivity. Mainly driven by striatal dysfunction in the brain, disrupted reward processing is a key characteristic in multiple types of addiction [33].

#### Psychosocial consequences

Early onset of cannabis use (<15 years of age) and frequent use in early adolescence are associated with lower educational achievement [34–36]. Low educational success includes early school dropout, lower rates of participation in university education and fewer academic degrees. Educational success is negatively associated with the age onset of cannabis use. There are inconsistent and limited data on antisocial behavior and psychosocial harm (e.g., family, professional and economic problems) [34]. The risk of physical violence is increased in regular cannabis users, especially in those with severe mental disorders [37, 38]. Risk of physical dating violence, victimization and perpetration is increased in female adolescent cannabis users and higher among alcohol users [39].

#### Mood and anxiety disorders

Research is showing that there are some individuals who use cannabis to reduce symptoms of mood or anxiety disorders [40]. Regular cannabis use is associated with more depressive symptoms, and heavy cannabis use by youth and young adults predicts an increased risk of major depression [41]; twofold for cannabis misusers and threefold to fivefold for people with a CUD [42]. Regular cannabis use is associated with anxiety symptoms [41] and increased risk of anxiety disorders particularly during the withdrawal stage [43]. Emerging evidence found a non-significant association between chronic cannabis consumption and social anxiety disorders [44, 45] and another study showed that cannabis use did not impede recovery from panic disorder’ when treated with other medications or treatments [46]. Risk of anxiety disorders is twofold higher in people who misuse cannabis, threefold to fourfold higher in those with a CUD [42, 45]. Associations between cannabis use and depressive or anxiety disorders were mixed in the reviewed studies, and weaker when analyses account for study quality, a diagnosis rather than symptoms of anxiety and early life psychosocial factors [43, 47]. Frequent cannabis use was associated with bipolar disorder and greater bipolar symptom severity, such as mania, rapid cycling, and mixed episodes [47]. There is strong early evidence that recent cannabis use is associated with negative long-term symptomatic and treatment outcomes across anxiety or mood disorders [46]. The literature is heterogeneous and associations may not be causal. Heavier and more frequent cannabis use was associated with suicidal ideation and suicide attempts in adolescents and young adults [48, 49]. The increases in suicide observed may reflect more impulsive behaviours. The causal pathways and mechanisms underlying the association between cannabis use, cannabis use disorder and anxiety or mood disorders remain unclear [40].

#### Psychotic disorders

Meta-analyses of case–control and cohort studies report a robust association between cannabis use and the risk of a psychotic disorder, and a dose–response relationship between this risk and the frequency of cannabis use [16]. A multinational study in Europe and Brazil [50] found that daily use of high potency cannabis increased the risk of psychosis fivefold compared to non-users while daily use of low potency cannabis doubled the risk. Use less than once a week was not associated with a psychotic disorder risk (other than acute psychoses which can emerge even with first time consumption of high THC doses). The prevalence of daily cannabis use and high potency cannabis use was positively associated with the incidence of treated psychosis across study sites in Europe and Brazil. More recently, a study based on electronic record over 5 decades in the Danish population reported an increase in the incidence of schizophrenia associated with use of THC and CUD, predominantly among young males 20–30 years of age in whom cannabis use accounted for 30% of the cases [51].

Triangulation of evidence from genetically sensitive research designs [52] indicates that the observed association may reflect in part a causal effect of cannabis use on schizophrenia. The risks of cannabis use are more severe for people who have psychotic disorders. In the laboratory, administration of THC increases psychotic symptoms in people with schizophrenia [53]. A meta-analysis of longitudinal studies [54] found that continued cannabis use in people with psychosis is associated with an increased risk of relapse to psychosis and with longer hospital stays than in non-users [55].

#### Cannabis use disorder (CUD)

CUD refers to clinically significant impairment or distress associated with repeated cannabis use. Its symptoms include an inability to control use, persistent use despite harmful consequences, tolerance to the effects of cannabis, withdrawal symptoms, and craving [21]. Worldwide, 22 million people are estimated to meet criteria for CUD, similar in number to opioid use disorders (27 million people) [56]. It is estimated that 22% of people who ever use cannabis meet criteria for CUD, increasing to 33% in those who use weekly or daily [57]. Key risk factors include being male, of younger age [57] and using high-potency cannabis [58]. Increases in cannabis potency have been associated with a faster progression to first onset of symptoms [59]. Psychosocial treatments such as motivational enhancement therapy, cognitive behavioral therapy, and contingency management produce greater reductions in cannabis use than control treatments [60]. There are no approved pharmacotherapies for the treatment of CUD [61].

#### Pregnancy

There is consistent evidence that regular cannabis use during pregnancy increases the risk of maternal anemia and neonatal problems such as reduced birth weight and the need for intensive care treatment [62]. Emerging preliminary evidence suggests that prenatal cannabis exposure may have subtle yet enduring effects on memory and achievement in children and adolescents [63]. There is limited evidence that some problems may persist into adulthood, such as delinquency, substance use and abuse, memory deficits, and psychotic symptoms [64].

#### Respiratory system

There is consistent evidence of an association between acute cannabis smoking and bronchodilation and between regular cannabis smoking and respiratory symptoms of cough, wheezing, and phlegm [65]. It is unclear the extent to which these effects are due to cannabis alone or its combined used with tobacco or other confounders. THC vaping has been associated with pulmonary injury due to the contaminants in electronic cigarettes and vaping pens (e-cigarette, or vaping, product use associated lung injury or EVALI) [66].

#### Cancer

Three case–control studies investigated the association between cannabis use and development of testicular germ cell tumors [67]. The strongest association was found for non-seminoma development and weekly cannabis use. The evidence is inconclusive for testicular seminomas, and lung cancer, neck and head cancers because of the possible roles of tobacco use, and other confounders.

#### Cardiovascular risks

Effects of cannabinoids on cardiovascular parameters seem to be complex and differential. They affect vasculature and myocardium directly via specific receptors and exert indirect effects through the central and peripheral nervous system. THC acutely stimulates the cardiovascular system, increases heart rate and produces a change in blood pressure. This occurs primarily due to sympathetic nervous system activation and parasympathetic nervous system inhibition [68]. There are case reports of adverse cardiovascular risks in chronic cannabis users (e.g., myocardial infarctions, tachycardia, dysrhythmias, hypotension, and orthostatic hypotension) [68–70]. There is consistent evidence of a temporal association between the onset of cardiovascular events and both chronic and acute cannabis intoxication [71]. There is emerging evidence that cannabis use is associated with an increased risk of acute coronary syndrome, especially in those with a history of cardiovascular events. There is a strong temporal link between cannabis exposure and the occurrence of stroke (i.e., ischemic), including chronic use and acute intoxication with onset ranging from “24 h” to “days before preceding the events” [72]. Potential confounding effects of co-substance use (e.g., tobacco and alcohol) in studies need to be acknowledged. The endogenous cannabinoid system may also provide an array of potential benefits, particularly in the role of CBD as a neuroprotector for stroke [73]. Rigorous studies supporting these positive effects are missing [72].

#### Hyperemesis syndrome

Cannabis can cause recurring bouts of nausea, vomiting and intense abdominal pain amidst other acute gastrointestinal symptoms that are severe enough to prompt users to seek medical help [23]. These cyclical episodes occur with prolonged, high-dose cannabis use and are alleviated by hot baths and showers and abstinence from cannabis [74]. The mechanism are not well understood but the disruption of the endocannabinoid system is a possible contributing factor.

#### Brain alterations

Case–control neuroimaging studies of the brain with high-resolution provide early evidence for altered brain integrity in brain regions with a high density of CB1 receptors in heavy cannabis users [75]. Specifically, chronic cannabis users have smaller volumes of the orbitofrontal cortex, a brain region implicated in motivation, and the hippocampus, a brain area implicated in stress, learning and memory [32]. Cannabis users and people intoxicated with THC had altered function in the same regions and in brain striatal areas implicated in reward processing. These alterations were more consistently observed in persons with a history of chronic cannabis use [76].

#### Mortality

Risk of death due to cannabis toxicity is very low, but there is a link between cannabis use at high doses and lethal motor vehicle collisions, cardiac pathophysiology and possibly suicide [17, 77, 78]. It is unclear whether mortality is related to cannabis use, predisposing factors or combinations thereof.

## Efficacy and safety of cannabis-based medicines

Due to their pharmacological properties, cannabis or cannabinoids are used for medical purposes (Table 1) [79, 80]. The literature on therapeutic effects of cannabis is limited to studies of delta-9-tetrahydrocannabinol (THC) and cannabidiol (CBD).

**Table 1:** Cannabis or cannabinoids used for medical purposes [79, 80]

Source	Commercial name	Indication
Synthetic delta-9-tetrahydrocannabinol (THC)^a^	Marinol, Syndros (dronabinol)	Anorexia with weight loss in AIDS wasting syndrome
Synthetic cannabinoid similar to delta-9-tetrahydrocannabinol (THC)	Cesamet and Canemese (nabilone)	Anorexia with weight loss in AIDS wasting syndrome Nausea and vomiting associated with chemotherapy, usually after previous treatments have failed
Plant based delta-9-tetrahydrocannabinol (THC) & cannabidiol (CBD)^a^	Sativex (nabiximols)	Muscle spasticity resulting from multiple sclerosis
Plant derived cannabidiol (CBD)^a^	Epidiolex	Seizures associated with Lennox-Gastaut syndrome or Dravet syndrome in patients 1 years of age and older Seizures associated with tuberous sclerosis complex in patients 1 year of age or older
Cannabis preparations	Raw cannabis, magistral preparations, standardized cannabis preparations	No approved indication

^a^Approved by FDA in the USA

### Chronic pain

Chronic pain includes neuropathic pain, fibromyalgia, rheumatoid arthritis and any pain in one or more anatomical region that persists or recurs for longer than 3 months and is associated with significant emotional distress or functional disability [20]. There is limited and inconsistent evidence that cannabinoids reduce chronic non-cancer pain by at least 30% but not more than 50% [81]. Cannabinoids may alleviate neuropathic pain in some patients [82], but there is insufficient evidence for other types of chronic pain [83, 84]. There is weak evidence for patient-perceived benefits [85]. Studies of the efficacy of cannabinoids for chronic pain treatment have usually been short-term (<12 weeks, partly only a few days) [86]. Cannabinoids have been administered as an oromucosal spray and combined with approved analgesics (e.g., opioids, anticonvulsants, or antidepressants) [82]. Mild to moderate central nervous or gastro-intestinal side effects are more common with cannabinoids than with placebo but severe side effects are rare [13]. Cannabinoids are recommended as second or third-line medications for chronic pain [87]. A large number of patients need to be treated to achieve a clinical benefit [81] and they are costly for patients [88]. There is also evidence that tolerance to the analgesic effects of cannabinoids develops with repeated use, which might limit their long-term benefits [89]. Medical cannabinoids may improve sleep in people living with chronic pain, but the magnitude of benefit is small [90].

### Muscle spasticity

Some cannabinoids (nabiximols, dronabinol, oral and oromucosal THC and THC/CBD, medicinal hemp) have small beneficial effects on muscle spasticity, primarily in patients with multiple sclerosis and possibly in those with spinal cord injuries and motor neuron disease. Benefits were more evident on patient than on physician ratings of muscle spasticity [91, 92]. Cannabinoids may increase slightly treatment discontinuation due to adverse events, nervous system and psychiatric disorders [93]. Nabiximols are only recommended if other treatments are ineffective, and a 4-week trial produces at least a 20% reduction in spasticity-related symptoms [88].

### Chemotherapeutically induced nausea and vomiting (CINV)

CINV is one of the few indications for which cannabinoids (dronabinol, nabilone, levonantradol, nabiximols) are approved by regulatory agencies such as the FDA in the USA. As an adjuvant treatment, they showed higher antiemetic effects than placebo or conventional anti-emetics [94]. These findings suggest that cannabinoids as promising therapeutic option for complicated CINV conditions (e.g., break-through, anticipatory or refractory). It should be pointed out here that newer antiemetics superseded many of the older antiemetics that cannabinoids were compared against. Only one RCT has compared cannabinoids to a guideline medication (ondansetron) for CINV prevention [95] and none compared them to new generation drugs (5-HT3- or NK1 antagonists or neuroleptics) [94].

In cancer anorexia cachexia syndrome patients’ cannabinoid treatment did not significantly improve appetite, oral intake, or anorexia-related quality of life [96].

### Epilepsy

CBD has recently been approved as Antiseizure medications for children and adults. A meta-analysis [97] shows that CBD is moderately efficacious both as standalone and adjunct therapy with clobazam for controlling refractory epilepsy in patients with Dravet syndrome, Lennox-Gastaut syndrome, and tuberous sclerosis complex.

### Eye disease

Effects of CBD-based cannabinoids are heterogeneous. THC-based effects on intraocular pressure are short-timed and reduced by the development of tolerance. Safe and effective drugs are already available on the market [98].

### Mental disorders

To date, only very few studies tested the effects of medicinal cannabinoids to improve symptoms of mental disorders, e.g. opioid dependence, cannabis use disorder, schizophrenia and schizophrenic psychoses, depressive disorders, posttraumatic stress disorder, anorexia nervosa, Tourette’s syndrome, attention-deficit hyperactivity disorder [99], autism spectrum disorder [100, 101] or dementia [13, 15]. In these studies, THC and/or CBD were administered with other pharmacotherapies and/or psychotherapy [15]. The paucity of data does not allow any guidance on the therapeutic use of cannabinoids for treating mental disorders within a regulatory framework [102].

### Other medical conditions

The evidence for therapeutic benefit of cannabis-based medicine is weak or conflicting for other medical conditions (see eText Box 2). Due to the role of the ECS in modulating inflammation, there is considerable interest in the therapeutic potential of cannabinoids in the treatment of autoimmune diseases such as rheumatoid arthritis and inflammatory bowel disease [103].

**eText Box 2:** Efficacy of cannabis-based medicines: other medical conditions

*Multiple sclerosis*: Tremor and nocturia was not consistently improved by cannabinoids (nabiximols, oral cannabinoids) [109]
*Parkinson’s disease*: In 3 out of 4 studies cannabinoids did not improve symptoms or those of l-dopa-induced dyskinesia (CBD, THC/CBD, nabilon, SR141716) [110]
*Huntington’s chorea*: Three small trials (84 participants) tested cannabinoids (CBD, nabilon, nabiximols) [102]. Significant treatment effects were found for nabilone
*Dystonia*: Two trials (24 participants) indicated lack of evidence on the use of cannabinoid for dystonia [102, 110]
*Dementia*: There is uncertain evidence for the efficacy and tolerability of cannabinoids in dementia [111, 112]
*Gastrointestinal disorders*: No firm conclusions could be drawn on the benefits and side effects of cannabinoids in adults with active Crohn’s disease (3 small RCTs, *n* = 93 participants) [113] or ulcerative colitis (2 RCTs, *n* = 92 participants) [114]. In patients with irritable bowel syndrome, cannabis and cannabinoids did not produce clinical remission or reduce inflammation (15 nonrandomized studies, 5 RCTs) but patient-reported symptoms and quality of life were significantly improved [115]

### Safety

Medical uses of THC-based cannabinoids are associated with an increased risk of short-term adverse effects, but severe side effects are rare [13]. THC-based products may alter thinking and perception, produce dizziness, lightheadedness and sedation particularly in older adults [104]. CBD is not intoxicating and has fewer safety concerns than THC, but there is the potential for liver toxicity, drug-drug interactions, and there is poor regulatory oversight of CBD products [105]. THC and/or CBD increase SSRI concentrations in adolescents, and coadministration of CBD and SSRIs increases the risk of cough, diarrhea, dizziness, and fatigue [106]. A systematic review recently estimated the prevalence of cannabis use disorders in people who use medicinal cannabis is estimated at 25% (CI: 18–33%) [107]. This risk could be higher in people with chronic non-cancer pain, mental health or substance use disorders [107]. An overview of adverse events reported in medicinal cannabis studies, medicinal cannabis product information, and post-marketing adverse event data can be found elsewhere [108].

## Conclusions

Cannabis is often used recreationally for its intoxicating effects and may have acute adverse health effects and impair the ability to safely drive a vehicle. More intense and prolonged patterns of use are linked with impaired: (1) educational attainment, (2) physical health (e.g., brain alterations, respiratory and cardiovascular risks, fetal damage, hyperemesis syndrome), (3) mental health (e.g., motivation, CUD, psychosis, mood and anxiety disorders), (4) suicide and mortality.

The increasing potency of cannabis products over the last few decades may increase the risk of addiction, impaired cognitive performance and mental disorders. It also appears to have increased the prevalence of overdosing, requiring emergency medical care. Though, the literature is sparse the evidence suggests that increased potency (higher THC concentrations) increases the acute and chronic adverse effects of cannabis.

Confounding of cannabis use by tobacco and other drug use complicates the interpretation of evidence on the harmful long-term health effects of cannabis smoking. There is debate about the extent to which the association between cannabis use and health risks is causal. Case–control and cohort studies are subject to unmeasured confounding—a limitation which may be overcome using genetically sensitive designs. More rigorous data is needed on health outcomes associated with increased cannabis potency, diversity of cannabis products and new routes of administrations.

Evidence on the therapeutic benefits of cannabis is limited to a few conditions and cannabinoids have been approved as therapeutics for a handful of indications. Strongest evidence for therapeutic benefit has been shown for CBD as an adjunctive treatment for intractable epilepsy in children and adults. THC may have benefits in improving spasticity, chronic pain, and chemotherapy-induced nausea and vomiting. The evidence base is inconclusive or too small to give treatment recommendations for neuropsychiatric disorders and other medical conditions. A major challenge in clinical trials is the absence of “standard” cannabinoids. The multiple preparations tested (e.g., dronabinol, Sativex spray, smoked cannabis flower) include different cannabinoids (e.g., THC, THC/CBD, CBD). Varied modes of use complicate standardized dosage and comparability in intra- and interindividual effects. Cannabinoids are also commonly used in addition to standard medications. This makes it difficult to estimate the effects of “pure” cannabinoids and drug-drug interactions, as seen for THC and/or CBD and SSRIs in adolescent patients. Cannabinoids are commonly well tolerated. In comparison with placebo, they have more adverse effects, which are transient and not severe in most cases. The metabolism of cannabinoids varies greatly between subjects in time course and quantity, so the same dose of cannabinoid can deliver different levels of bio-available cannabinoids to different patients, producing varied therapeutic effects and non-response.

### Implications for policy

Those countries that decide to legalise recreational adult use of cannabis should regulate production, sales and promotions of cannabis use in ways that delay cannabis use among youth and minimize the regular use of cannabis products with high doses of THC. They should also consider funding more robust research on the short and long-term effects of cannabinoids. Public education about the risks and the benefits of medical and nonmedical cannabis use should be a high policy priority. In locations where the recreational purchase of cannabis is legal, the general public and especially vulnerable individuals (e.g., pregnant or breastfeeding women, people with health problems) should also be informed about the risks of self-medication. Clinicians administering cannabinoids need to be mindful of patients’ cardiovascular risks and vulnerability to stroke. Doctors should take into account the risk of cannabis use disorder and other adverse effects when prescribing cannabis or cannabinoids for mental or physical health problems.

## Supplementary Information

Below is the link to the electronic supplementary material.

Supplementary file1 (PDF 100 KB)
