# Supplementary material for: Cannabis, cannabinoids and health: a review of evidence on risks and medical benefits
Source: Eur Arch Psychiatry Clin Neurosci. 2024 Sep 19;275(2):281–92. doi: 10.1007/s00406-024-01880-2 (PMC11910417; doi:10.1007/s00406-024-01880-2)
Supplement: Supplementary file 1 — Supplementary file1 (PDF 100 KB) [file 406_2024_1880_MOESM1_ESM.pdf]

eText box 2; Efficacy of cannabis-based medicines: Other medical conditions

Multiple sclerosis: Tremor and nocturia was not consistently improved by cannabinoids (nabiximols, oral cannabinoids) (Koppel et al., 2014).

Parkinson's disease: In 3 out of 4 studies cannabinoids did not improve symptoms or those of L-dopa-induced dyskinesia (CBD, THC/CBD, nabilon, SR141716) (Andrzejewski, Barbano, & Mink, 2016).

Huntington's chorea: Three small trials (84 participants) tested cannabinoids (CBD, nabilon, nabiximols) (Lim et al., 2017). Significant treatment effects were found for nabilone.

Dystonia: Two trials (24 participants) indicated lack of evidence on the use of cannabinoid for dystonia (Andrzejewski et al., 2016, Lim et al., 2017).

Dementia: There is uncertain evidence for the efficacy and tolerability of cannabinoids in dementia (Bahji, Meyyappan, & Hawken, 2019; Bosnjak Kuharic et al., 2021).

Gastrointestinal disorders: No firm conclusions could be drawn on the benefits and side effects of cannabinoids in adults with active Crohn's disease (3 small RCTs, n=93 participants) (Kafil, Nguyen, MacDonald, & Chande, 2018a) or ulcerative colitis (2 RCTs, n=92 participants) (Kafil, Nguyen, MacDonald, & Chande, 2018b). In patients with irritable bowel syndrome, cannabis and cannabinoids did not produce clinical remission or reduce inflammation (15 nonrandomized studies, 5 RCTs) but patient-reported symptoms and quality of life were significantly improved (Doeve, van de Meeberg, van Schaik, & Fidder, 2021).

eReferences

Koppel, B. S., Brust, J. C., Fife, T., Bronstein, J., Youssof, S., Gronseth, G., & Gloss, D. (2014). Systematic review: efficacy and safety of medical marijuana in selected neurologic disorders: report of the Guideline Development Subcommittee of the American Academy of Neurology. Neurology, 82(17), 1556–1563. https://doi.org/10.1212/WNL.0000000000000363

Andrzejewski K, Barbano R, Mink J. Cannabinoids in the treatment of movement disorders: A systematic review of case series and clinical trials. Basal Ganglia. 2016;6(3):173-181.

Lim K, See YM, Lee J. A Systematic Review of the Effectiveness of Medical Cannabis for Psychiatric, Movement and Neurodegenerative Disorders. Clin Psychopharmacol Neurosci. 2017;15(4):301-312. https://doi.org/10.9758/cpn.2017.15.4.301

Bahji, A., Meyyappan, A. C., & Hawken, E. R. (2020). Cannabinoids for the Neuropsychiatric Symptoms of Dementia: A Systematic Review and Meta-Analysis. Canadian journal of psychiatry. Revue canadienne de psychiatrie, 65(6), 365–376. https://doi.org/10.1177/0706743719892717

Bosnjak Kuharic, D., Markovic, D., Brkovic, T., Jeric Kegalj, M., Rubic, Z., Vuica Vukasovic, A., Jeroncic, A., & Puljak, L. (2021). Cannabinoids for the treatment of dementia. The Cochrane database of systematic reviews, 9(9), CD012820. https://doi.org/10.1002/14651858.CD012820.pub2

Kafil, T. S., Nguyen, T. M., MacDonald, J. K., & Chande, N. (2018). Cannabis for the treatment of Crohn's disease. The Cochrane database of systematic reviews, 11(11), CD012853. https://doi.org/10.1002/14651858.CD012853.pub2

Kafil, T. S., Nguyen, T. M., MacDonald, J. K., & Chande, N. (2018). Cannabis for the treatment of ulcerative colitis. The Cochrane database of systematic reviews, 11(11), CD012954. https://doi.org/10.1002/14651858.CD012954.pub2

Doeve, B. H., van de Meeberg, M. M., van Schaik, F. D. M., & Fidder, H. H. (2021). A Systematic Review With Meta-Analysis of the Efficacy of Cannabis and Cannabinoids for Inflammatory Bowel Disease: What Can We Learn From Randomized and Nonrandomized Studies?. Journal of clinical gastroenterology, 55(9), 798–809. https://doi.org/10.1097/MCG.0000000000001393
